# Clarifying Misconceptions About School‐Based Health Care

**DOI:** 10.1111/josh.13543

**Published:** 2025-01-13

**Authors:** Irwin Mary Kay, Sara Bode, Daniel Skinner

**Affiliations:** ^1^ School Health Services Nationwide Children's Hospital Columbus Ohio USA; ^2^ Department of Pediatrics The Ohio State University Columbus Ohio USA; ^3^ Department of Social Medicine Ohio University, Heritage College of Osteopathic Medicine Dublin Ohio USA

**Keywords:** partnering with parents and other caregivers, primary care, reproductive health, school‐based clinics

## Abstract

**Background:**

To health professionals working in American school‐based health centers, the benefits of school‐based health programs are obvious. The philosophical warrant for this work has been reasserted for over 70 years. And yet, the divisiveness of health and health care in the U.S. has led to questions and even concerns about the appropriateness of co‐locating healthcare services in schools.

**Contributions to Practice:**

We address three common misconceptions to provide an accurate depiction of the work school‐based health centers do. The misconceptions we address are: 1. that education and health care should be separate; 2. that parents and guardians alone are responsible for their children's health; and 3. that school‐based health centers cut out parents and guardians from involvement in their children's health.

**Implications for School Health Policy, Practice, and Equity:**

Misconceptions about school‐based health centers undermine the important work being done in these centers, including the pursuit of improved child health outcomes. Clarifying these misconceptions is of the utmost importance.

**Conclusions:**

It is important to understand and actively address common misconceptions about school‐based health centers. Doing so can help leverage the opportunities these centers and programs present.

**Abbreviation**
SBHC: School‐Based Health Center

## 1 Introduction

To health professionals working in American school‐based health centers (SBHCs), the benefits of school‐based programs are obvious. The philosophical and scientific warrants for this work were first issued by the World Health Organization more than 70 years ago and have been reasserted continually [1, 2, 3]. And yet, as would be expected given the divisiveness of health and health care in contemporary American society, school health personnel have encountered questions and even concerns about the appropriateness of co‐locating healthcare services in schools; some challenge the type of services offered as well as parental involvement in school‐based medical appointments. In this commentary we address three common misconceptions to provide an accurate depiction of the work and to illuminate the core principles of school‐based health programs.

## 2 Three Misconceptions About SBHCs

### 2.1 Misconception 1: Education and Healthcare Should Be Separate

Personnel are sometimes called to defend school health's foundational premise that schools are uniquely situated to address child health needs. The inequities that underlay school education funding raise legitimate questions about whether schools should really be taking on an additional role when the education of children is already challenging enough. While schools rarely bear sole financial responsibility for SBHCs, they indirectly spend limited resources to ensure the success of these clinics. For example, schools commonly provide premium space for SBHCs within school buildings and frequently dedicate staff time and resources to support clinic implementation and integration [4].

This concern misses the nature of the relationship between child health and education. As scholars have documented, child health is a foundation of educational attainment, not only in terms of educational performance, but in being able to attend school at all [5]. In this regard, investments in school health do not detract from, but support educational investments.

In fact, both institutions have long been entwined. Schools, for example, have long been concerned with health promotion through health education classes and the presence of school nurses [6]. To address the mental health needs of students, schools commonly invest in variations of support inclusive of school counselors, social workers, psychologists and prevention programming. A closer look reveals that school‐based health is an extension, to a logical next step, of a commitment to health promotion that has always been part of schools' missions [7].

### 2.2 Misconception 2: Parents and Guardians Are Responsible for Their Children's Health

Critics sometimes charge that SBHCs are providing services to children who should be seeking care at traditional health facilities [8]. A related criticism holds that school health is compensating for a lack of attentiveness on the part of parents and guardians to their children's needs. The criticism holds, in other words, that parents and guardians should be responsible for meeting their children's health needs [9].

There are several reasons why these criticisms are misguided. First, they underestimate the challenges parents and guardians face today related to transportation barriers [10], and a paucity of pediatricians with capacity to offer after hours care for working parents and who accept Medicaid [11, 12]. Second, they fail to take an equitable approach to health promotion as traditional health facilities are sometimes unable to accommodate the barriers mentioned above that prevent patients and families from accessing needed care.

Research has evolved our understanding of the meaning of “equitable access” in health care [13]. Equitable access is about more than proximity, type of services, and office hours. Many students are connected to primary care providers, but a growing segment of the student population is not [14]. These students lack access due to barriers such as poverty, a lack of transportation, parental work schedules, lack of trust in the medical system, health insurance challenges, and health literacy, among other things.

Third, as the COVID‐19 pandemic reminded us, public health is about meeting patients where they are and understanding their contexts and needs. For example, during the pandemic, schools became a place to stop the spread of infection by teaching students and their families how to prevent transmission. Additionally, schools were leveraged as sites of care to test and immunize millions. It may be tempting to say that patients should “just see a doctor,” but the complex sociology of healthcare relationships teaches us that while patient‐parental responsibility is a part of the conversation, health professionals and the institutions within which they practice have an obligation to actively accommodate patients and their families [15, 16]. Leveraging schools as sites of care as a public health and health equity strategy can be an effective way to accommodate patients and families.

At the same time, chronic absenteeism in school, which has become a growing problem across the U.S. [17], can be considered a public health issue given the frequency that unmet healthcare needs contribute to frequent absences. It is well documented in the literature that students experiencing chronic absenteeism are more likely to have poor health outcomes. The interdependence of wellbeing and school attendance calls for flexibility on the part of both schools and health providers. SBHCs provide a powerful tool for addressing this problem [1, 18].

### 2.3 Misconception 3: SBHCs Cut Out Parents and Guardians

A related but separate misconception points to the other side of the parental coin, namely that school‐based programs undermine parental autonomy and decision‐making. As we might expect from a healthcare environment that is increasingly politically polarized, some critics are concerned that SBHCs will lead to medical decision‐making without appropriate parental involvement, especially—but not only—concerning immunizations, mental health care and reproductive and sexual health.

Experience with SBHCs paints a different picture than these fears suggest. More than a decade ago, the Centers for Disease Control published useful guidance for ensuring meaningful parental and guardian involvement in school health [19]. While many SBHCs do not require parents to attend appointments with their child, providers still obtain parental consent for treatment. Additionally, parents of children seen in SBHCs are always welcome to attend appointments and if attending in‐person presents a barrier, parents can participate via speaker phone, FaceTime, or telehealth. Further, school‐based providers are accustomed to calling parents before appointments to obtain an updated medical history—and often call parents again to discuss the appointment as well as send home an after‐visit summary document. While immunizations, mental health care, and sexual and reproductive health may be politically contentious, and school health centers do typically provide vaccinations, mental health and reproductive health services, most parents and guardians are not only accepting of the support SBHCs provide, but they are grateful that caring professionals are available to help their children [20, 21].

Far from carrying out this work without parents and guardians, in most situations SBHCs collaborate with them. In fact, an underappreciated aspect of SBHCs' work is the translational and educational work they do with families, not only about children's health needs, but health care access more generally. SBHC personnel spend a great deal of time communicating, educating, and supporting parents and guardians so they can play active roles in the promotion of their child's health.

## 3 SBHCs: A Reality‐Based Approach to Community Health

Given the rancorousness that sometimes accompanies health in our current American political moment, it makes sense that the rise of SBHCs would generate strong reactions. After all, changes to the delivery of care—especially in spaces such as schools, with established traditions and meanings—requires effective communication and careful planning. There is good reason to be skeptical and seek assurances that parental and guardian concerns, clinical standards, and educational norms are being respected. SBHCs nullify most of the concerns identified in this perspective. It's important that health advocates address them directly.

Despite the expanding influence of SBHCs, it is important to remind critics that schools have always been involved in care delivery and have always partnered with parents and guardians to address children's health needs. SBHCs are less of a radical shift in health care than an extension of a longstanding tradition.

At the same time, we should not downplay the scale of what SBHCs demand of us. School health programs are complex endeavors. As these programs pursue their ambitious goals of contributing meaningfully to improvements in population health, they are positioned not only as champions, but disruptors. It takes an endless stream of meetings and daily communications to keep these programs moving in a way that can best serve the children, their families, and their schools. Only if all stakeholders—within schools and within healthcare institutions alike—understand the institutional and public health history that brought us to school‐based health will we be able to focus on the hard work ahead.

## Conflicts of Interest

The authors declare no conflicts of interest.
